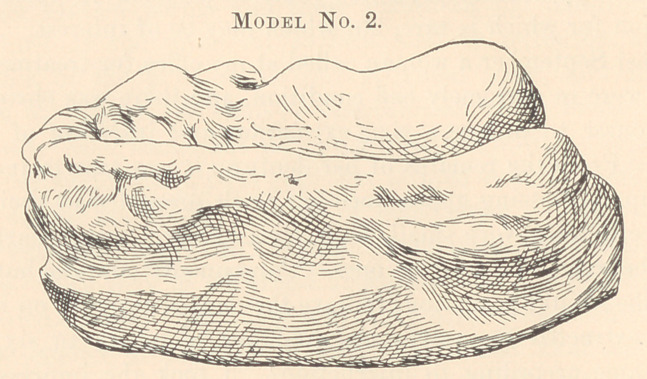# An Unusual Operation

**Published:** 1902-01

**Authors:** W. R. Howard

**Affiliations:** Newport, R. I.


### AN UNUSUAL OPERATION.

BY W. R. HOWARD, D.D.S., NEWPORT, R. I.

THE opportunity presented itself to perform an operation, the occasion for which is rare, but which may be of interest.

Last September a woman called at my office for treatment. Her teeth were in extremely bad condition, and there was obviously but one course to pursue,—viz., extraction and insertion of artificial teeth. From the remains of her denture, it was easy to judge what an unprepossessing appearance her teeth presented at their best,—the teeth being very small, with spaces of at least a quarter of an inch between the anterior ones. Consequently, it was but natural that she should be anxious to have the dentist improve on nature.

I extracted the teeth, and told her to return in six months. She came according to instructions. I took the impression, and started to make the upper denture, but on inserting the trial plate I found it absolutely impossible to obtain any effect that could be tolerated, on account of the protuberance of the alveolus in the anterior portion of the mouth. I think any one can get an idea of the impossibility of the case by a glance at Model No. 1.

What to do I was at a loss to know; but it occurred to me that it ought to be possible and practicable to remove quite a portion of the alveolar process. I searched through reference books for a precedent, but could find none. I then consulted Dr. Brackett (Professor of Pathology at Harvard Dental School) and Dr. Darrah, a local surgeon of ability, and we decided that there could be no objection to the course I suggested; accordingly, we decided to carry it out.

Model No. 1.

The operation was done at the patient's home. She was laid on a long table and ether administered; then with a surgeon's knife I made a clean incision from cuspid to cuspid on a line with the natural position of the teeth clear through to the bone. Then with

Model No. 2.

a periosteal elevator I pushed back the gum and periosteum, completely exposing the alveolar process for some distance both lingually and labially. With a pair of alveolar forceps I made an incision through the alveolus at the median line from a quarter- to a half-inch in depth, and with that as a starting-point, using surgeon's bone-clippers, removed the alveolus to about the same depth to each of the cuspids. At Dr. Brackett's suggestion, I had the dental engine at hand, with a variety of mounted carborundum stones, and found it but a few moments' work to grind smooth any roughness which remained after using the bone-clippers, and which would have very much retarded the process of healing. The gum was then replaced and trimmed, allowing a sufficient amount for shrinkage, and four sutures of catgut made to hold it in place.

I saw the patient the following day, and she seemed to be progressing favorably, with very little soreness of the gums. I told her to return when it seemed to be thoroughly healed and free from tenderness, and, to my surprise, she was back in just two weeks from the day of the operation. I proceeded to make an artificial denture without any gum in front, and succeeded very much to her satisfaction as well as my own.

Model No. 2 shows the mouth after operating, though the actual case ought to be seen to realize the great change that was made in her appearance.

## Figures and Tables

**Model No. 1. f1:**
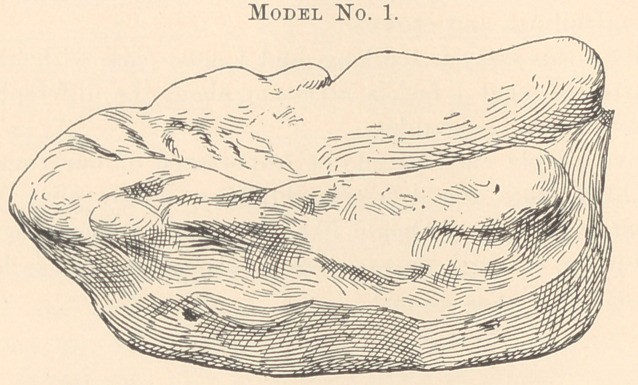


**Model No. 2. f2:**